# Design of a Ultra-Stable Low-Noise Space Camera Based on a Large Target CMOS Detector and Image Data Analysis

**DOI:** 10.3390/s22249991

**Published:** 2022-12-18

**Authors:** Chao Shen, Caiwen Ma, Wei Gao

**Affiliations:** 1 Xi’an Institute of Optics and Precision Mechanics of Chinese Academy of Sciences, Xi’an 710119, China; 2 University of Chinese Academy of Sciences, Beijing 100049, China

**Keywords:** CMOS detector, space camera, low readout noise, ultra-stable, image data analysis

## Abstract

To detect faint target stars of 22nd magnitude and above, an astronomical exploration project requires its space camera’s readout noise to be less than 5e^−^ with long-time working stability. Due to the limitation of satellite, the traditional CCD detector-based camera does not meet the requirements, including volume, weight, and power consumption. Thereby, a low-noise ultra-stable camera based on 9 K × 9 K large target surface CMOS is designed to meet the needs. For the first time, the low-noise ultra-stable camera based on CMOS detector will be applied to space astronomy projects, remote sensing imaging, resource survey, atmospheric and oceanic observation and other fields. In this paper, the design of the camera is introduced in detail, and the camera is tested for several rounds at −40 °C; it also undergoes further testing and data analysis. Tests proved super stability and that the readout noise is lower than 4.5e^−^. Dark current, nonlinearity and PTC indicators meet the requirements of the astronomical exploration project.

## 1. Introduction

Space cameras are applied to high-precision photometry, resource census, terrain mapping, and military reconnaissance. With the continuous development of space remote sensing technology, space cameras have broad application prospects in astronomical observation, environmental monitoring, atmospheric observation, oceanic observation, and earth radiation measurement.

**Figure 1:**
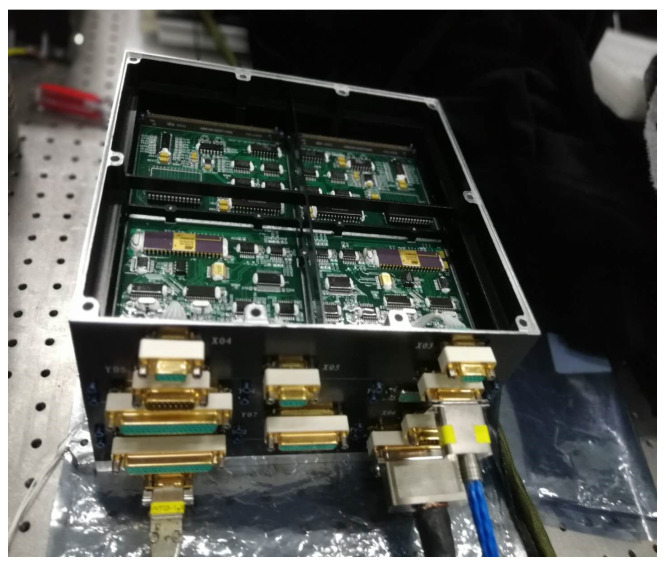
Electronics of the SVOM astronomical camera.

At present, most international astronomical observation projects use CCD (charge-coupled device) [1,2] detectors. CCD image sensors convert optical signals to analog current signals directly. After amplification and analog-to-digital conversion, current signals achieve the acquisition, storage, processing, reproduction, and transmission of images. The CCD image sensor has many advantages, such as a small size, a light weight, low power consumption, and low operating voltage. However, its hardware drive circuit is complex, with high costs and power consumption. The electronics of the Chinese–French SVOM (Space Variable Objects Monitor) astronomical camera developed based on CCD detector are shown in Figure 1.

The camera is designed based on a 2 K × 2 K CCD detector. The camera electronic consists of five circuit boards; each of which has an area of 260 mm × 230 mm. The camera system readout noise is 6e^−^.

CMOS (Complementary Metal Oxide Semiconductor image sensors) [3,4,5] sensors use the CMOS process, which is most commonly used in general semiconductor circuits. Thereby, the convenience of integrating peripheral circuits (such as ADC [6], CDS, Timing generator or DSP) [7] into the sensor chip can save the cost of peripheral chips. In addition, the CMOS sensor image acquisition method is active, the charge generated by the photodiode will be directly amplified by the transistor output. In contrast, the passive acquisition method of the CCD sensor requires additional voltage to move the charge in each pixel. In addition to the difficulty in power management and circuit design, the high-drive voltage required by CCD sensors makes them far more energy intensive than CMOS sensors.

With the rapid development of CMOS circuit technology, it provides a good condition for the production of CMOS image sensors, with a large target surface, a high dynamic range and fast readout.

There is no low-noise CMOS detector to realize space-borne astronomical projects in China, and there is no mature large-target low-noise CMOS detector (photosensitive area exceeds 6 K × 6 K) for satellite-borne astronomical projects in the world so far.

However, an astronomical exploration project needs to detect stars of 22nd magnitude and fainter target stars, which puts forward higher requirements on the imaging target surface, readout noise, and stability of space cameras. The main technical indicators of the space camera are shown in Table 1.

From Table 1, we can see this project requires its space camera’s readout noise to be less than 5e^−^ with long-time working stability. In addition, due to the limitation of satellite, the traditional CCD detector-based camera does not meet the requirements, including volume, weight, and power consumption.

Thereby, based on the actual requirements of this astronomical project, to realize the project, we first designed an ultra-stable low-noise [8,9,10,11,12] camera prototype based on a 9K*9K large target surface CMOS detector, which is newly developed by a Chinese company. The camera prototype includes a low-noise CMOS driver board, a FPGA control board and a low-noise secondary power supply board; the area of each circuit board is 230 mm × 210 mm. Compared with the developed SVOM camera based on the CCD detector, the prototype has the advantages of low power consumption, small volume, light weight, and low readout noise. This camera will be subsequently applied to ultra-high precision photometry, remote sensing imaging, environmental detection and other fields.

## 2. Design of Camera System

**Figure 2:**
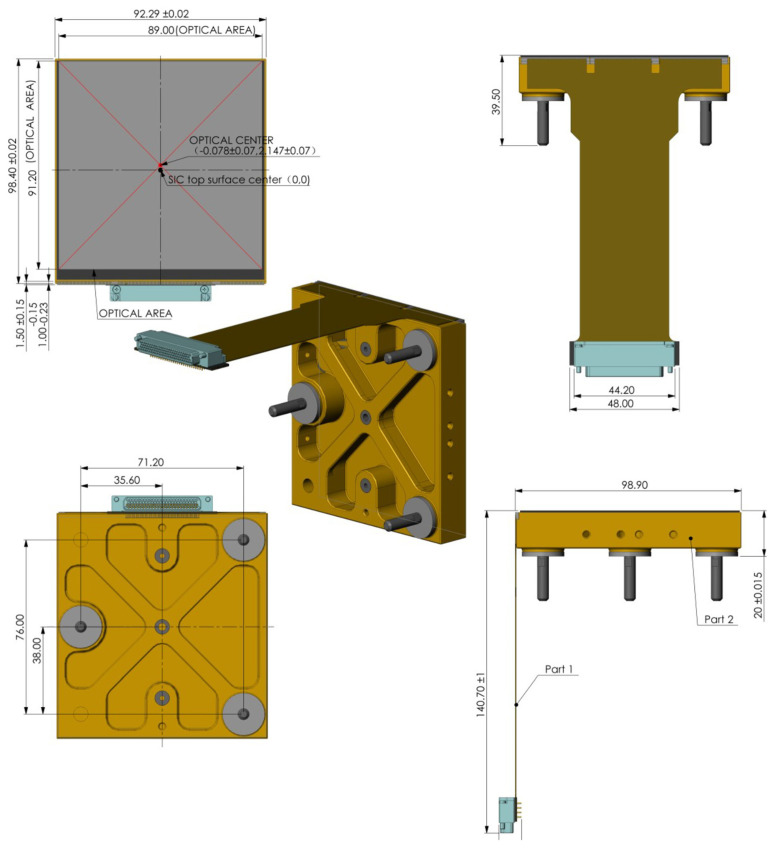
9 K × 9 K CMOS detector model.

The space camera is designed based on a 9 K × 9 K CMOS detector, this detector model is shown in Figure 2.

The detector has its own flexible cable with 100 pins, including 27 single-timing control signals, four pairs of differential data signals, a pair of differential input clock signals, a pair of differential clock output signals, five power supply signals and 12 bias power supply signals. The detailed technical specifications of the detector are shown in Table 2.

**Figure 3:**
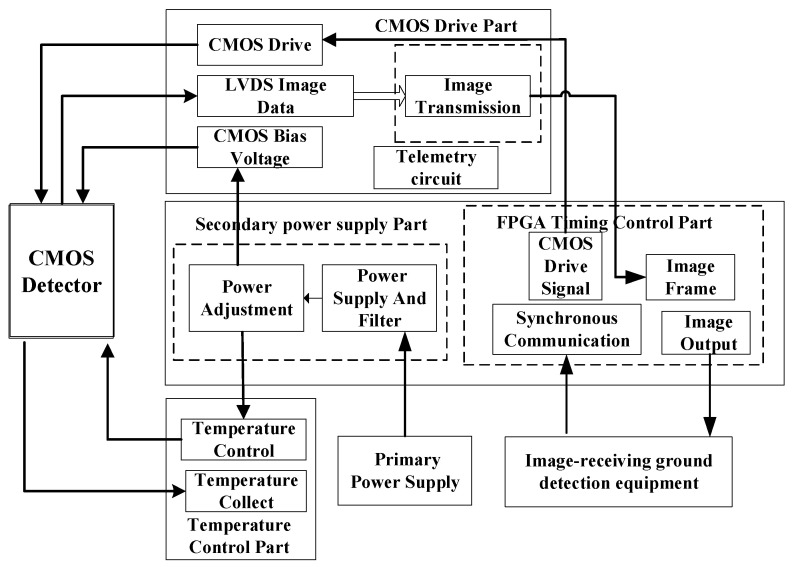
The camera prototype signal flow diagram.

As shown in Table 2, the number of pixels, pixel size and resolution of the CMOS detector meet the requirements of camera indicators. The camera prototype signal flow diagram is shown in Figure 3.

From Figure 3, we can see that the camera system consists of the CMOS detector part, the CMOS drive part, the FPGA [13] timing control part, the secondary power supply part, the temperature control part and the image-receiving ground detection equipment part.

The main functions of the camera are listed below:

(1) Generating power supplies required by the circuit through DC/DC converters and LDO-linear-regulated power supplies;
(2) Providing drive signals for CMOS detectors;
(3) Acquiring, superimposing, and caching digital images. Packaging working parameters into the image data and transmitting them down to the image-receiving ground detection equipment through the fiber optic interface after completing the target image element superimposition selection;
(4) Setting working parameters, switching working mode, and controlling the working state according to remote control commands received;
(5) Completing the acquisition and output of telemetry data.

### 2.1. Secondary Power Supply Design for Camera

**Figure 4:**
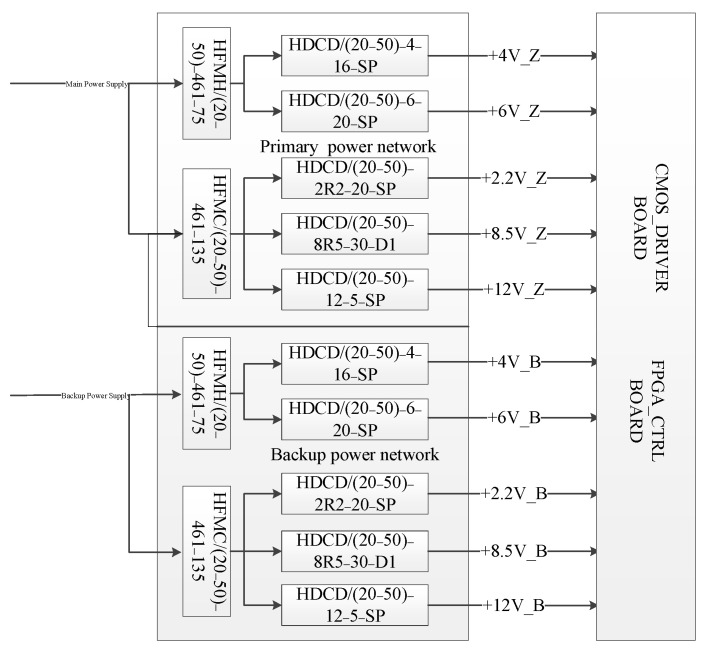
The camera secondary power supply block diagram.

The secondary power supply provides five power supplies for the CMOS driver board and the FPGA control board. The secondary power supply block diagram is shown in Figure 4.

As shown in Figure 4, in order to improve device reliability, we use primary backup design, receiving the 30 V bus power supply, respectively, and generating +4 V, +6 V, +2.2 V, +8.5 V and + 12 V five power supplies through the DCDC converter. To keep the noise low, fully isolated DC/DC modules are applied to each power supply. EMI filter and LC filter circuits are added at the input end of the DC/DC module, and a π-type filter circuit is added at the output end. The reduction of ripple and noise from the output to the CMOS driver board allows the camera system to achieve a low-noise readout.

### 2.2. Design of CMOS Detector Power Supply, Bias Voltage and Driver

**Figure 5:**
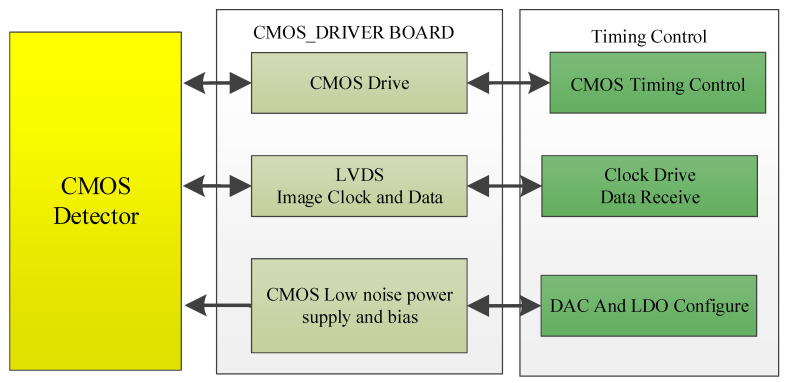
The signal flow block diagram of the CMOS driver board.

The signal flow block diagram of the CMOS driver board is shown in Figure 5.

From Figure 5, we can clearly know that the CMOS driver board provides driving signal, input clock, low-noise power supply and bias voltage for the detector, and completes the reception of clock and image data output by the CMOS detector.

The CMOS detector power supply and bias voltage required for the operation of this CMOS detector are shown in Table 3.

According to the task indicator requirements, the CMOS camera readout noise was ≤5e^−^. The readout noise of the camera system is mainly composed of detector noise, power supply noise, bias noise, and quantization noise. The detector noise is determined by its performance and needs to be optimized for power supply, bias, and other noise to meet the task requirements.

Many factors need to be considered in order to lower noise and achieve high-speed readout, including power supply, bias, drive, etc. The noise must be guaranteed to be extremely low, while taking into account the interference and crosstalk caused by a high-speed readout. The reference source for bias and detector signal processing must ensure high stability and noise below 100 μV. Low-noise power supply is used in the circuit design. The secondary power supply can be supplied with less than 50 μV noise through the tertiary LDO. The high-stability and low-noise bias is achieved by a high-stability low-temperature efficient voltage reference source, a precision DAC combined with a low-noise op-amp. The operating temperature of the camera during operation must be stabilized at −40 °C ± 0.1 °C for a long time.

In addition, the drive signal must ensure signal integrity, control the CMOS drive signal waveform, and control its rising and falling edges. Within the allowable range, rising and falling edges should be as slow as possible, and preferably without overshoot. Reduce the high-frequency ponent to ensure signal integrity to avoid interference feedthrough. In addition, the PCB layout and wiring will also have an impact on the noise.

**Figure 6:**
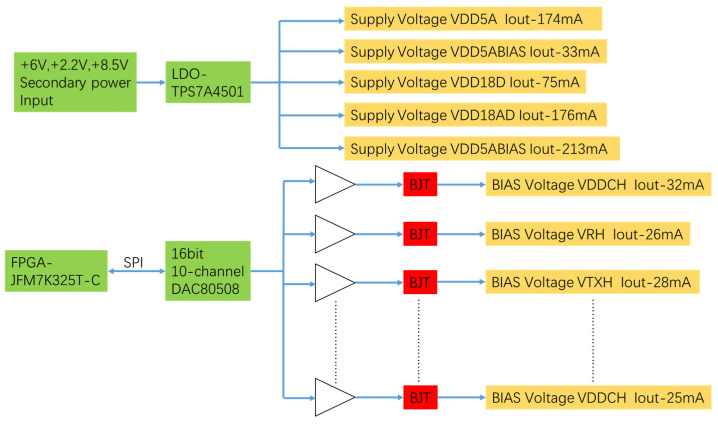
Low-noise power supply and low-noise bias block diagram of CMOS detector.

Considering the above factors, low-noise design is very difficult, so the implementation is difficult. The low-noise power supply and the low-noise bias block diagram of the CMOS detector is shown in Figure 6.

**Figure 7:**
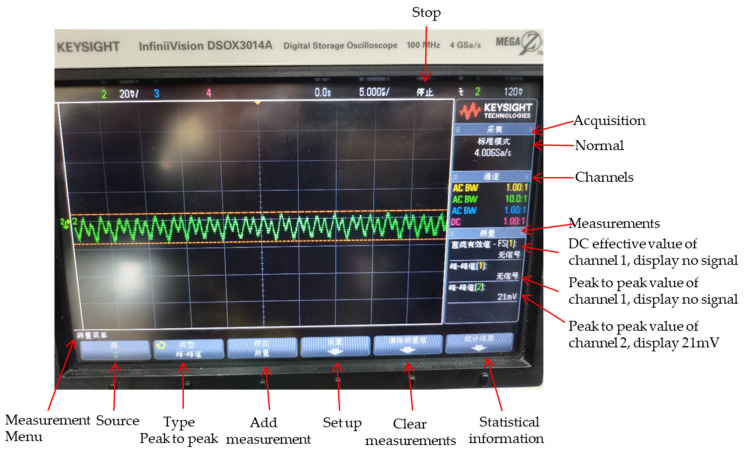
The ripple of input 6 V power supply for generate VDD5A.

In the actual design, because VDD18D, VDD18AD, VDD5A, VDD5ABIAS and VDDSF require large currents, the LDO chosen to supply the power is TI’s TPS7A4501 chip, which has a high output voltage accuracy of 1.15%, an excellent load transient response, and can ensure stable output when the load changes. The power supply ripple rejection ratio (PSRR) is 68 dB at 1 kHz. The ripple passing through this LDO is 2512 times than that before the input. The ripple of the LDO input 6 V power supply for generate VDD5A is shown in Figure 7.

From Figure 7, we can see that the ripple of the input 6 V power supply is 21 mV. The ripple is less than 10 uV after passing through this LDO. The detector operates at 5 V, and the FPGA output is at 3.3 V level, a level conversion chip is needed to generate the 36-way drive signal level required for the normal operation of the detector.

The detector needs twelve low-noise bias voltages for operation, which is provided by high-precision DAC with low-noise operational amplifier and triode. The DAC uses TI’s DAC80508, which has 16-bit resolution, 2 ppm/°C temperature drift, and ±1 LSB linearity, allowing for high accuracy and stable output. The precision reference source selects ADI’s ADR4550, whose temperature drift is 2 ppm/°C, its output voltage accuracy is ±0.02%, and the output voltage noise is at 0.1 Hz to 10 Hz is 1.25 uVp-p.

**Figure 8:**
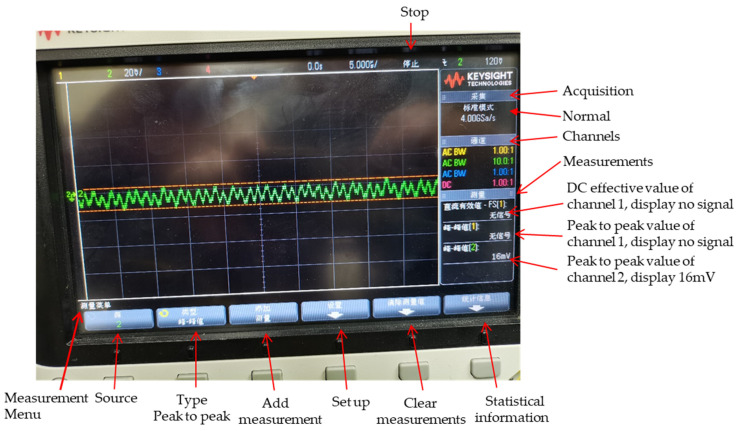
The ripple for positive input of the operational amplifier for generating VDCH bias voltage.

The operational amplifier is ADI ADA4084-4S, the op-amp noise at 1 KHz is 3.9 nV/√Hz, and its PSRR can reach more than 70 dB, meaning that the ripple through the op-amp is 3162 times higher than that before the input; the device in 10,000 h voltage offset is less than 3 uV. The ripple for positive input of the operational amplifier for generating VDCH bias voltage is shown in Figure 8.

From Figure 8, we can see that the ripple for positive input of the operational amplifier for generating VDCH bias voltage is 16 mV; the ripple is less than 10 uV after passing through this operational amplifier. The triode mainly plays the role of the current expansion.

### 2.3. Design of FPGA Control

**Figure 9:**
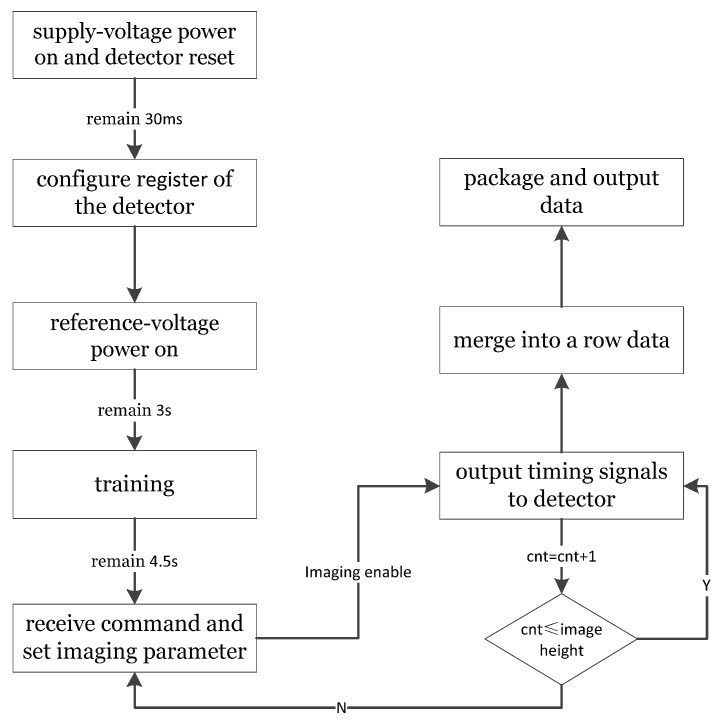
The system workflow diagram.

The FPGA control board generates all the timing signals required by the CMOS detector, completes data transmission via optical fiber, and completes external communication. The timing control unit acts as the communication hub of the CMOS camera, establishes an LVDS bus communication link with Star Service, realizes synchronization control, power control and status telemetry, generates the CMOS timing drive signal and control signal, and manages data cache. The FPGA receives the digital image signal from the CMOS detector and transmits it to the image-receiving ground detection equipment. The system workflow diagram is shown in Figure 9.

## 3. Data Collection and Test Result Analysis

### 3.1. Test at Room Temperature

**Figure 10:**
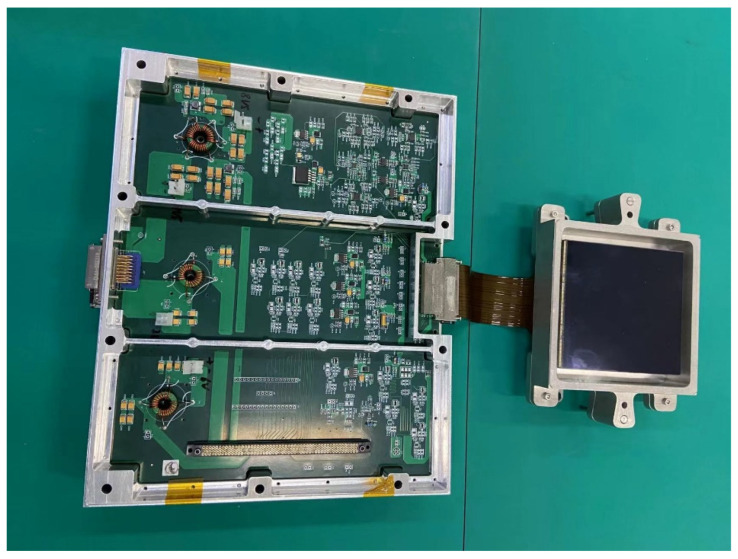
The space camera prototype.

According to the requirements of the project, we developed a prototype space camera based on 9 K × 9 K CMOS detector. The space camera prototype is shown in Figure 10. This prototype consists of one 9 K × 9 K CMOS detector, one CMOS driver board, one FPGA control board, and one secondary power supply board.

**Figure 11:**
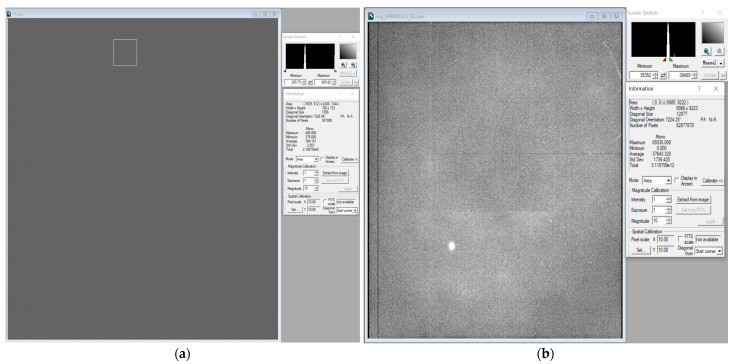
Test at room temperature. A dark and a flat image. (**a**) Dark image; (**b**) Flat image.

At room temperature, we tested a dark and a flat image, the image is shown in Figure 11.

**Figure 12:**
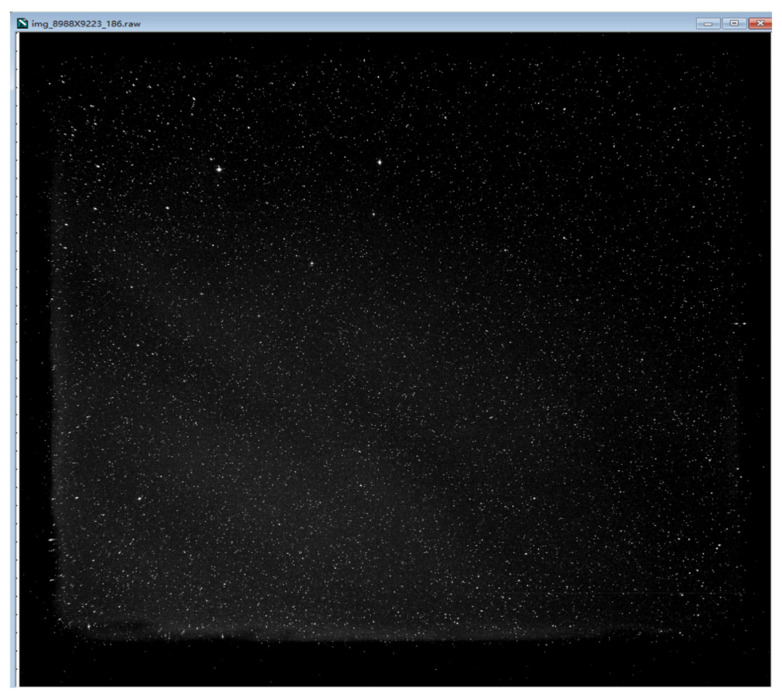
The star map image used this CMOS camera take.

The main test result at room temperature is that the readout noise is about 4.4e^−^, the reading interval is 2.94 s, so the fps (frames per second)) [14] is 0.34. We also used this camera to take a star map image; the star map image is shown in Figure 12.

### 3.2. Test Results under Low-Temperature and Image Data Analysis

To eliminate the influence of dark current on readout noise, we built a camera test system environment. The system contains image-receiving ground inspection equipment, vacuum Dewar equipment, high-precision temperature control system, and a monochromator integrating sphere. The detector is placed in the Dewar for cooling, and the camera electronic is placed outside the Dewar, which work in the laboratory environment. The camera electronic connected with the detector through flexible cables.

**Figure 13:**
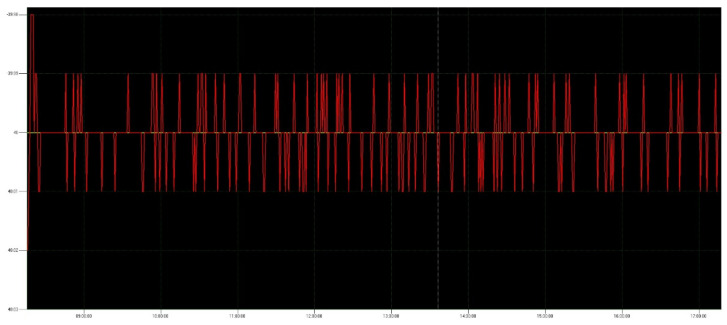
Temperature control system accuracy test result.

The CMOS detector is first installed in the Dewar and the evacuation equipment is turned on. After the vacuum level reaches below 10^−3^ Pa, the TEC [15] controller is turned on to cool the detector assembly until the temperature reaches −40 °C. In order to reduce the impact of temperature drift and ensure the thermal stability of the detector assembly, the temperature control accuracy is required to be ±0.1 °C. The test time of the temperature control system exceeds 8 h each time, the result is shown in Figure 13.

**Figure 14:**
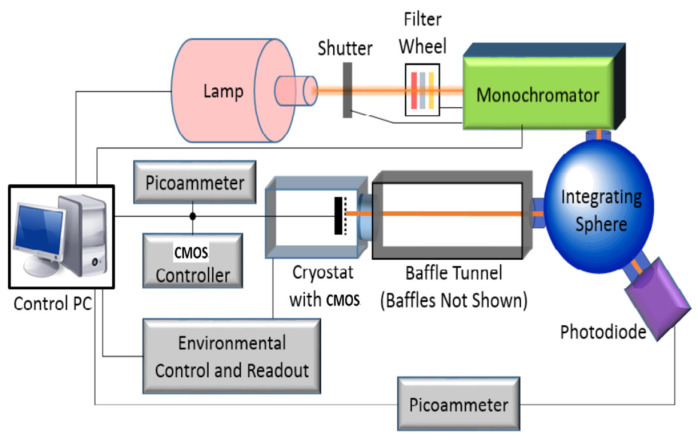
Schematic diagram of camera test system.

**Figure 15:**
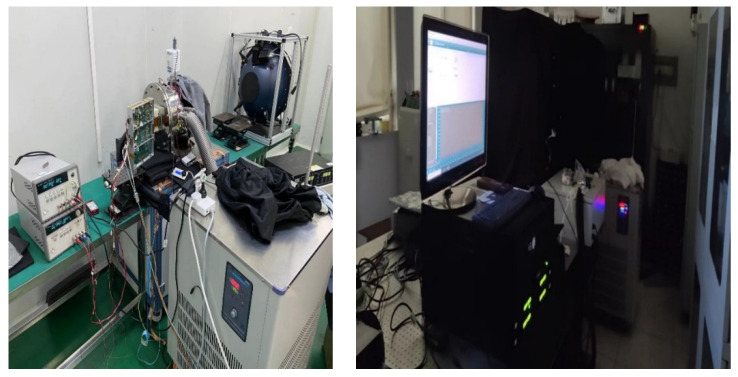
Physical picture of camera test system.

Form Figure 13, we can see that the temperature control accuracy is less than ±0.1 °C, meeting the requirement. The dark field test starts when the temperature reaches −40 °C ± 0.1 °C. The integrating sphere is turned on to provide a uniform light source for the detector during the flat-field test. Image-receiving ground detection equipment is used to collect the image data generated by the CMOS camera and control the camera. The schematic and physical diagrams of the test environment are shown in Figure 14 and Figure 15, respectively.

In order to realize the low-noise and ultra-stable test of the camera, from 28 June 2022 to 4 August 2022, we tested 10 times in the same environment, which is shown in Figure 14. Before each test, the detector should be cooled to −40 °C ± 0.1 °C. First, collect 50 bias images, then turn off the camera, then power on the camera, and collect 50 bias images the second time. Then, set 5 s, 10 s, 50 s, 100 s, 200 s, 400 s, and 800 s exposure times for the camera and collect five dark field images for each exposure time. Finally, open the integrating sphere, set the integrating sphere to the stable mode, ensuring that the light source error is within 1%. Change the exposure time, and collect the flat field image of the camera from the bias to full well capacity. Each test takes more than 8 h.

According to the collected image, gain [16], readout noise [17], nonlinearity [18,19,20], photon transfer curve (PTC) [18,19,20] and dark current [18,19,20] have been analyzed in detail. The above indicators are analyzed according to the EMVA (European Machine Vision Association) standard 1288 release 4.0 general, so detailed calculation methods for each indicator will not be explained. The test result is shown in Table 4 and Table 5, Table 5 is part of Table 4. Table 4 gives the test time, gain and readout noise test results. Table 5 gives the nonlinearity, PTC, dark current test results. The two tables also give the median value of indicator at the bottom.

The camera has been tested 10 times in 2 months; each test takes more than 8 h. Within 2 months, the change of test ambient temperature and humidity may affect the camera and test equipment. The readout noise stability calculation equation is given by:

(1) $$std_{r}=\frac{1}{N_{k}}((\sum_{k=1}^{N_{k}}(r_{k}-r_{m})/r_{m})k\times 100\%$$

where $std_{r}$ is the stability of readout noise, $r_{k}$ is each time test result, $r_{m}$ is the median value of 10 times test results, k is number of test. According to Equation (1), the readout noise stability calculate result is 0.474%, better than 0.5%. The calculation results show that the camera is, basically, not affected by the change of test ambient temperature and humidity within 2 months; the readout noise stability of the camera is excellent.

As below, we present the test results of various indicators of the camera in 28 June 2022, 6 July 2022 and 19 July 2022, respectively. The test results of other dates are similar to those of the three days, so they will not be listed one by one.

**Figure 16:**
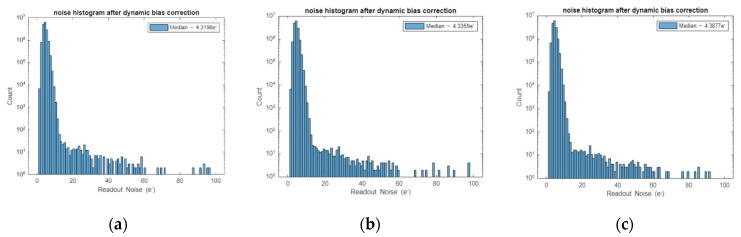
The readout noise test result of the camera. (**a**) 28 June 2022 test result; (**b**) 6 July 2022 test result; (**c**) 19 July 2022 test result.

The test result of the camera readout noise is shown in Figure 16. We can see the camera readout noise is less than 4.5e^−^.

**Figure 17:**
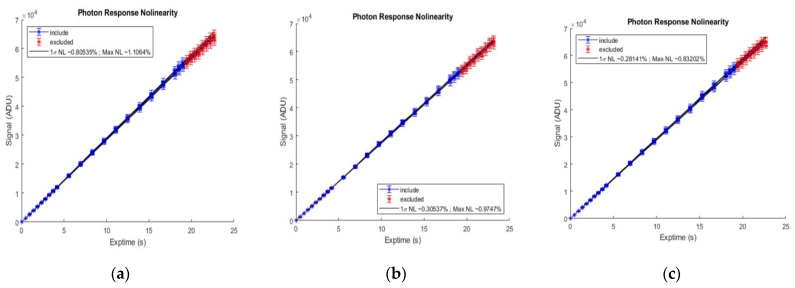
The nonlinearity test result of camera. (**a**) 28 June 2022 test result; (**b**) 6 July 2022 test result; (**c**) 19 July 2022 test result.

The camera nonlinearity test result is shown in Figure 17. It can be seen from the figure that the camera’s nonlinearity is less than 1% at 10–90% FW.

**Figure 18:**
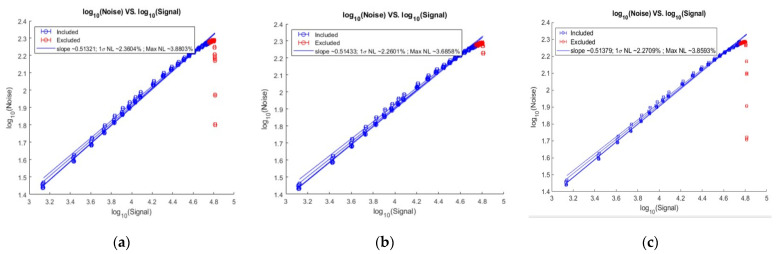
The PTC test result of the camera. (**a**) 28 June 2022 test result; (**b**) 6 July 2022 test result; (**c**) 19 July 2022 test result.

The camera PTC test result is shown in Figure 18. Under the condition of 10–90% FW, the PTC is less than 3%.

**Figure 19:**
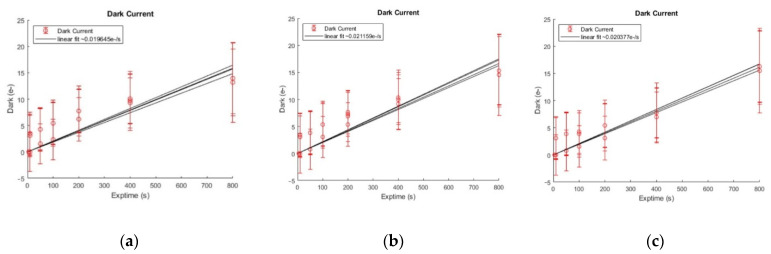
The dark current test result of the camera. (**a**) 28 June 2022 test result; (**b**) 6 July 2022 test result; (**c**) 19 July 2022 test result.

The dark current test result of the camera is shown in Figure 19. We can see the dark current is less than 0.1e^−^/(pix·s) at −40 °C low temperature.

## 4. Conclusions

The design of a large target ultra-stable low-noise space camera based on 9K × 9K is illustrated. The development of the camera prototype includes: building the high-precision vacuum cryogenic testing system and completing a camera performance test at room temperature and −40 °C low-temperature performance. The test results show that the camera has the advantages of low readout noise and super stability, and the dark current, PTC, nonlinearity indicators meet the needs of astronomical exploration projects. This camera can meet the needs of astronomical exploration projects, expanding the application of the space camera based on the CMOS detector in space astronomical observation field. It solves the disadvantages of large volume high-power consumption, and the high cost of traditional CCD cameras. In the future, this camera will also be widely used in high-precision photometry, remote sensing imaging, resource survey, atmospheric and oceanic observation, as well as other fields.

## Figures and Tables

**Table 1:** Main technical indicators of the space camera.

Order Number	Indicator Term	Technical Indicator
1	Pixel number (uint: mm)	≥6144 × 6144
2	Pixel size	10 um × 10 um
3	Readout noise(e^−^/pixel/frame)	≤5e^−^
4	Resolution	16 bit
5	Dark current e^−^/(pix·s)@ −40 °C	≤0.1e^−^/(pix·s)
6	Nonlinearity (10–90% FW)	≤1%
7	PTC (10–90% FW)	≤3%

**Table 2:** Technical specifications of the CMOS detector.

Number	Parameter	Value
1	Photosensitive area	89.00 mm × 91.20 mm
2	Pixel size	10 μm × 10 μm
3	Number of active pixels	8900 (H) × 9120 (V)
4	Shutter type	Rolling shutter
5	Pixel clock rate	Up to 15.625 MHz @ 16-bit
6	Output format	5 pairs of LVDS in total: - 4 for pixel data; - 1 for DDR clock;
7	Data rate	500 Mbps @ 2 pairs of LVDS running at 250 MHz
8	Max Frame rate	0.34 fps @ 2 pairs of LVDS
9	Full well capacity (FWC)	91.7 ke-
10	Dynamic range	84.5 dB
11	Dark current	0.00373e^−^/s/pix @ −70 °C
12	Peak QE	97.11% @ 610 nm
13	Power consumption	1.4 W @ full resolution & full speed
14	Operation temperature	−85 °C to +50 °C

**Table 3:** Supply and bias power of CMOS detector.

Name	Classification	Voltage Typical Value (V)
VDD5A	Supply Voltage	+5.0
VDD5ABIAS	Supply Voltage	+5.0
VDD18D	Supply Voltage	+1.8
VDD18AD	Supply Voltage	+1.8
VDDSF	Supply Voltage	+6.0
VDDCH	Bias Voltage	+4.7
VDDCL	Bias Voltage	+2.0
VRH	Bias Voltage	+5.7
VRL	Bias Voltage	0.0
VTXH	Bias Voltage	+4.7
VTXL	Bias Voltage	0.0
VSH	Bias Voltage	+5.0 or +6.0 (Change according to exposure time)
VBSH	Bias Voltage	+5.0
VBG	Bias Voltage	+1.25
VPC_LOAD	Bias Voltage	+1.0
VRAMP_INIT	Bias Voltage	+4.7
VRAMP_PC	Bias Voltage	+4.55

**Table 4:** The 10 times gain and readout noise test results of the camera in the same environment.

Date	Gain(e−/ADU)	Readout Noise (e−/pixel/frame)
28 June 2022	1.52540	4.38770
1 July 2022	1.52630	4.32070
4 July 2022	1.52150	4.32380
5 July 2022	1.52850	4.31770
6 July 2022	1.53070	4.33590
7 July 2022	1.53470	4.31730
8 July 2022	1.53390	4.32360
11 July 2022	1.52330	4.34720
19 July 2022	1.53060	4.31980
4 August 2022	1.53690	4.39340
Median	1.52918	4.33839

**Table 5:** The 10 times Nonlinearity, PTC and Dark current test results.

Nonlinearity (10–90% FW)	PTC (10–90% FW)	Dark Current e−/(pix·s) −40 °C
~0.805%	2.360%	0.01964
~0.647%	2.045%	0.01473
~0.574%	2.255%	0.01634
~0.485%	2.286%	0.02093
~0.281%	2.260%	0.02111
~0.826%	2.372%	0.01688
~0.632%	2.331%	0.02069
~0.835%	2.203%	0.01854
~0.305%	2.271%	0.02030
~0.837%	2.360%	0.02008
~0.630%	2.271%	0.01886

## Data Availability

Not applicable.
